# Practical Lessons from Theoretical Models about the Somitogenesis

**Published:** 2007-05-28

**Authors:** Aitor González, Ryoichiro Kageyama

**Affiliations:** Institute for Virus Research, Kyoto University, and Japan Science and Technology Agency, CREST Kyoto, Japan

**Keywords:** Theoretical models, mouse somitogenesis, Hes7 oscillation, Notch signaling, Fgf signaling, Wnt signaling

## Abstract

Vertebrae and other mammalian repetitive structures are formed from embryonic organs called somites. Somites arise sequentially from the unsegmented presomitic mesoderm (PSM). In mice, a new bilateral pair of somites arise every two hours from the rostral PSM. On the other hand, cells are added to the caudal side of the PSM due to cell proliferation of the tail bud. Somite formation correlates with cycles of cell-autonomous expression in the PSM of genes like *Hes7*. Because the somitogenesis is a highly dynamic and coordinated process, this event has been subjected to extensive theoretical modeling. Here, we describe the current understanding about the somitogenesis in mouse embryos with an emphasis on insights gained from computer simulations. It is worth noting that the combination of experiments and computer simulations has uncovered dynamical properties of the somitogenesis clock such as the transcription/translation delays, the half-life and the synchronization mechanism across the PSM. Theoretical models have also been useful to provide predictions and rigorous hypothesis about poorly understood processes such as the mechanisms by which the temporal PSM oscillations are arrested and converted into an spatial pattern. We aim at reviewing this theoretical literature in such a way that experimentalists might appreciate the resulting conclusions.

## Introduction

**Figure1:**
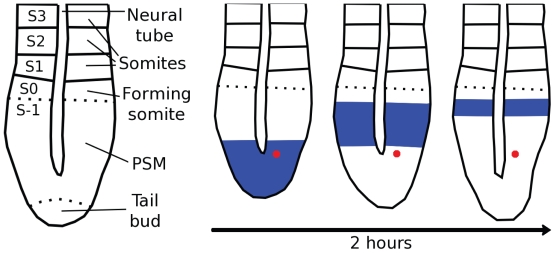
(**a**) This drawing represents the tail of a mouse embryo. The somites and the neural tube can be recognized morphologically. To visualize the boundary of the forming somite, molecular markers such as *Mesp2* expression are needed. Finally, the tail bud refers to the tip of the tail, where extensive proliferation occurs. S−1, S0, S1,… are terms commonly used to refer to the somites. S0 is the forming somite, S1 the newest somite, etc. (**b**) Waves of cell-autonomous gene expression (blue) spread from the caudal to the rostral PSM, with a period that correlates with that of the somite formation. Cells do not actively move. However, only tail bud cells extensively proliferate, so that the distance of a cell (red) to the tail bud increases whereas the distance to the somite decreases.

The somites are embryonic organs that develop to repetitive structures such as vertebrae (sclerotome), striated musculature of the trunk and limbs (myotome), and subcutaneous tissue (dermatome) (Drews, 1995). Somite formation is initiated at around the 8th embryonic day (E8), where the (unsegmented) presomitic mesoderm (PSM) is sequentially sectioned along the rostral-caudal axis (Figure 1(a)). This segmentation process occurs at a pace of about one pair of somites every 2 hours. On the other hand, new cells are added to the PSM because of cell proliferation in the tail bud. This has the effect that although cells are rather immobile to each other, they move relative to the tail bud and the somite (Figure 1(b)). Furthermore, the somite formation correlates with cycles of cell-autonomous gene expression that spread from the tail bud to the rostral PSM border with a periodicity equal to that of the somitogenesis (Palmeirim et al. 1997).

These highly coordinated and dynamic processes harden the intuitive understanding of the somitogenesis. Therefore, computer simulations have been often employed to gain insights into the underlying processes (reviewed by Schnell et al. 2002; Baker et al. 2003; Freitas et al. 2005). Unfortunately, conclusions from theoretical models pass sometimes unnoticed by biologists because of the difficulty of the underlying mathematics. Here, we summarize conclusions from recent theoretical works, so that we complement recent reviews written from a more experimentalist perspective (Aulehla and Pourquié, 2006; Gridley, 2006; Freitas et al. 2005; Aulehla and Herrmann, 2004; Rida et al. 2004). We will refer to mouse data if not stated differently.

## Classical models about the somitogenesis

Some authors have proposed Turing-like models for the somitogenesis. In Turing-like models, there are two reacting and diffusing substances, an activator and an inhibitor (reviewed by Miura and Maini, 2004; see a biological example by Sick et al. 2006). Under appropriate parameter values, these Turing-like substances create stripes from a homogeneous medium as observed during the somitogenesis (Meinhardt, 1982, 1986; Kaern et al. 2004; Schiffmann, 2004). This model has been validated in a chemical reactor (Kaern et al. 2004), but no evidence has been provided in vivo for such Turing-like substances in the case of the somitogenesis.

At the end of the eighties, it was found that a single heat shock applied to a developing chick embryo induces regular somite and skeletal anomalies separated from each other by 6–7 somites (Primmett et al. 1988). The time delay for the formation of 6–7 somites corresponds to one cell cycle, which led to the proposal of the cell-cycle model. In the cell-cycle model, the segmentation pace is controlled by the cell cycle, which oscillates to some degree in synchrony in PSM cells. This model further defines two phase points of the cell cycle. Cells reaching the second phase point signal to cells between the first and second phase point to form a somite together (Stern et al. 1988; Primmett et al. 1989). This model has been formalized mathematically by several authors (Polezhaev, 1992; Collier et al. 2000; McInerney et al. 2004). However, no further correlation has been found between the cell cycle and the somitogenesis period.

The third classical model, the clock-and-wavefront model, states that all cells in the PSM undergo synchronously a state oscillation under the control of a molecular clock. In parallel, there is a wavefront of maturation that moves in the rostral-caudal direction and arrests the clock of the PSM cells in one of the oscillating states (Cooke and Zeeman, 1976; Cooke, 1981). This model gained popularity after observations that the chick *c-hairy1* gene oscillates with a period that is identical to that of the somitogenesis (Figure 1(b)) (Palmeirim et al. 1997; Cooke, 1998).

## Coupled negative feedback loops drive the oscillations

Many genes oscillate in the PSM, notably members of the Notch, Wnt and Fgf pathways, as shown by microarrays (Dequéant et al. 2006). Some of these cyclic genes are required for proper segmentation of the PSM (summarized by Gridley, 2006).

**Figure 2:**
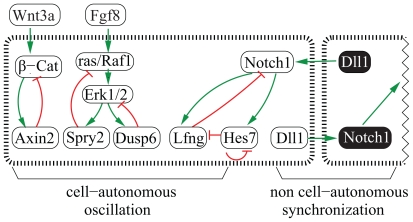
At least, five negative and one positive intracellular feedback loops act in the mouse PSM. The five negative feedback loops are thought to drive the oscillations: *Hes7* on itself; *Lfng* and Notch1; β-Cat and Axin2; ras/Raf1, Erk1/2 and Spry2; Erk1/2 and Dusp6. On the other hand, the interactions from Hes7 to Lfng, Lfng to Notch1 and Notch1 to Hes7 create an intracellular positive feedback loop, which probably couples the oscillations within the Notch pathway. Furthermore, an intercellular positive feedback loop via the Notch pathway has been hypothesized to synchronize neighboring cells. Green and red arrows represent positive and negative interactions, respectively. White and black gene products represent neighboring cells.

The oscillation of some cyclic genes relies on negative feedback loops (Figure 2). In the PSM, Lunatic fringe (Lfng) glycosylates thus inhibiting the Notch1 receptor, whose activation is required for the Lfng expression. This negative feedback loop results in the oscillation of Lfng expression (Morales et al. 2002; Dale et al. 2003; Morimoto et al. 2005).

The gene products of *Hairy and enhancer of split 1* and *7* (*Hes1/7*) are also targets of the Notch1 pathway, and directly bind to their promoters to inhibit their own expression, which results in their oscillations (Jouve et al. 2000; Hirata et al. 2002; Bessho et al. 2003). Furthermore, Hes7 is required for the Lfng oscillations, which provides a further link between the Lfng/Notch1 and the Hes7 feedback loops (Bessho et al. 2001a, 2003).

The Fgf pathway is active mainly in the caudal PSM (see below). In the Fgf pathway, the expressions of *Dusp6* and *Spry2* oscillate in phase with the previous Notch pathway genes (Dequéant et al. 2006). In chick, the expressions of *Dusp6* and *Spry2* are induced by the diphosphorylation of the Erk1/2 proteins (Delfini et al. 2005). Dusp6 dephosphorylates and inactivates Erk1/2 thus creating a negative feedback loop with Erk1/2 (Figure 2) (Li et al. 2007, and references therein). Spry2 inhibits the activation of ras and Raf (reviewed by Mason et al. 2006), so that another negative feedback loop is formed by ras/Raf, Erk1/2 and Spry2 (Figure 2).

The Wnt pathway is also active mainly in the caudal PSM (see below) and some of its members oscillate in opposite phase to the Notch and Fgf pathway genes (Dequéant et al. 2006). The expression of *Axin2* depends on Wnt3a and inhibits the Wnt pathway by promoting together with GSK-3 the phosphorylation and degradation of β-Catenin (Liu et al. 2005; reviewed by Moon et al. 2002). This suggests that the Axin2 oscillation depends on a negative feedback (reviewed by Aulehla and Herrmann, 2004; Aulehla and Pourquié, 2006).

## The Fgf8, Wnt3a and Raldh2 gradients

There are three sources of asymmetry along the rostral-caudal axis of the PSM, two gradients of Fgf8 and Wnt3a spreading from the tail bud, and a gradient of Raldh2 (retinoic acid (RA) synthesizing enzyme) spreading from the somites (Aulehla et al. 2003; Dubrulle and Pourquié, 2004; Delfini et al. 2005; del Corral et al. 2003). The Fgf8 gradient requires Wnt3a activity (Aulehla et al. 2003).

*Fgf8* mRNA is synthesized in the tail bud, but becomes degraded when the cells move away from the tail bud. This *Fgf8* mRNA gradient is translated into a Fgf8 protein gradient, which maintains PSM cells in an undetermined state (Dubrulle et al, 2001; Aulehla et al, 2003; Dubrulle and Pourquié, 2004).

By contrast, Raldh2 is strongly expressed in the somites of chick PSM and promotes neuronal differentiation. Interestingly, it was found that *Fgf8* expression and Fgf8 signaling was shifted rostrally in RA deficient chick mutants. By contrast, Fgf8 was able to repress *Raldh2* expression (del Corral et al. 2003). This mutual repression of the Fgf8 and RA pathways results in a positive feedback loop that might sharpen the Fgf8 concentration threshold, where PSM cells switch from undetermined to determined states.

On the other hand, the connections between the gradient genes and the cyclic genes are not well known. We have already mentioned the dependence of Axin2 expression on the Wnt3a gradient. Recently, it has been shown in chick that Fgf8 and Wnt3a signaling are necessary for the cyclic expression of the *Snail* genes. Furthermore, overexpression of *Snail2* prevents the cyclic *Lfng* expression, which suggests another link between the gradients and the cyclic genes (Dale et al. 2006).

## Dynamical properties of the clock

**Figure 3:**
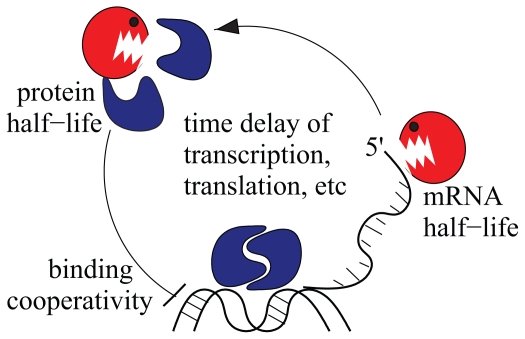
Three critical features have been suggested for the Hes1/7 oscillation: The characteristic time delays of eukaryotic transcription/translation, an appropriate short half-life of both mRNA and protein, and finally a well-defined cooperative binding (Hill) coefficient of the protein to its promoter, which might depend, for instance on the number of binding sites.

Among the best characterized segmentation clocks are the *Hes1/7* genes, whose product are able to bind and inhibit their respective promoters (Hirata et al. 2002; Bessho et al. 2001b). In the last years, the Hes1/7 negative feedback loops have been studied in detail both experimentally and computationally, and interesting dynamical properties have been uncovered (Figure 3).

Initially, it was believed that a one-gene negative feedback loop is unable to generate sustained oscillations (Hirata et al. 2002). However, computer simulations showed that a one-gene negative feedback loop generates sustained oscillations if the intrinsic time delays due to transcription, translation and nuclear transport are taken into account (Jensen et al. 2003; Lewis, 2003; Monk, 2003). Furthermore, in support to the delay hypothesis, it has been recently found in fibroblasts that the peak of the unprocessed *Hes1* mRNA is found 40 minutes after cell stimulation, whereas that of processed *Hes1* mRNA is found one hour after cell stimulation (Masamizu et al. 2006).

Several pieces of evidence have demonstrated that the half-life of the Hes1/7 proteins and *Lfng* mRNA plays a very important role for the oscillations (Chen et al. 2005). In the case of Hes1/7, the role of degradation was elegantly addressed by a combination of *in vitro*, *in vivo* and *in silico* methods. By arresting the protein synthesis in fibroblasts and plotting the decrease of Hes7 concentration, a half-life of 22.3 minutes was measured. Then, a knock-in mouse was generated where the Hes7 protein had a slightly longer half-life (30.3 minutes). This mouse showed a perturbed somitogenesis thus underlining the importance of the Hes7 half-life. Finally, computer simulations with the wild-type and mutant half-lives showed that only the wild-type Hes7 half-life results in sustained oscillation (Hirata et al. 2004).

The oscillation of Hes1/7 largely depends on binding to its promoter. The cooperativity of protein-DNA interactions determines how abrupt the promoter state changes from active to inactive and vice versa. The Hill coefficient gives a measure of such cooperativity. Computer simulations suggest that there exists a critical value of the Hill coefficient, under which the Hes1/7 negative feedback loop might not undergo sustained oscillations anymore (Monk, 2003; Barrio et al. 2006; Bernard et al. 2006; Zeiser et al. 2006). However, different factors influence the value of the critical Hill coefficient. A model of Hes7 interaction with its promoter indicates a value 2 for the critical Hill coefficient under the assumption that Hes7 interacts with a single binding site. By contrast, if it is assumed that Hes7 interacts cooperatively with three binding sites, the critical Hill coefficient value increases to 2.6 (Zeiser et al. 2006). Interactions with other cofactors appear to decrease the critical value of the Hill coefficient. In another model, the critical Hill coefficient fell down from 4.5 to 2.5 after the Hes1 cofactor transducine-like enhancer of split/Groucho-related gene product 1 (TLE) was taken into account (Bernard et al. 2006). The value of the critical Hill coefficient also falls down if the intrinsic stochasticity of cellular processes is included in the model (Barrio et al. 2006). Hence, the Hill coefficient seems to be an important parameter to achieve sustained oscillations though it remains to be proved experimentally.

## Synchronization of the oscillations

Mouse fibroblasts undergo oscillation of *Hes1* expression after serum shock. Western-blots of *Hes1* protein from the whole cell population suggested that the Hes1 oscillation is damped and arrested after eight hours (Hirata et al. 2002). Nevertheless, real-time imaging of individual cells has recently revealed that the oscillation continues in individual cells for longer than eight hours (Masamizu et al. 2006). This suggests that the dumping observed at the whole-population level arises from desynchronization of Hes1 oscillation in individual cells. This conclusion has been supported by stochastic computer simulations, where many individual cell trajectories were computed. Then, the arithmetic mean values were plotted at each time point, which showed a damped oscillation arising from individual oscillators canceling each other (Barrio et al. 2006).

In the PSM, neighboring cells oscillate in phase for long periods. This synchronization is lost in zebrafish mutants for Notch signaling (Jiang et al. 2000). Computer simulations have shown that a positive feedback loop between neighboring cells is sufficient to keep neighboring cells in phase (Figure 2) (Lewis, 2003; Horikawa et al. 2006; Masamizu et al. 2006). Experimental evidence has come from a recent set of experiments in zebrafish where PSM tissue from a donor was transplanted into another PSM host. After transplantation, the oscillation phase of the explants became synchronized with that of the host (Horikawa et al. 2006).

Along the rostral-caudal axis, there is a constant phase difference between the rostral and caudal halves of the PSM due to the slowing down of the oscillation (Figure 1(b)). To show the existence of a coupling mechanism along the rostral-caudal axis, the PSM was dissected, and the oscillations in the fragments were visualized by real-time microscopy. In this experiment, the dissected fragments easily lose their relative phase difference though the oscillations continue stably in each fragment (Masamizu et al. 2006). Two theoretical models have successfully reproduced the oscillation synchronization through the Notch pathway along the rostral-caudal axis. In addition, these models make interesting predictions about how the oscillation phase difference along the rostral-caudal axis arises. The first model for chick and mouse predicts that this phase difference arises from a gradient in the intercellular coupling strength (Cinquin, 2003). The second model for zebrafish predicts that a gradient of the concentration of the mRNA of the *hairy/Espl* gene *her13.2* causes the oscillation phase difference (Cinquin, 2007).

As mentioned above, members of the Fgf and Notch pathways oscillate in phase with each other, and in opposite phase to the members of the Wnt pathway (Dequéant et al. 2006). There is some coupling between these pathways as, for instance, disruption of the Wnt pathway disrupts the Lfng oscillations (Aulehla et al. 2003). Nevertheless, the connections are not clear yet. A candidate to couple the oscillations of the Wnt and Notch pathways is Nkd1, which oscillates in phase with Lfng oscillation and requires both Wnt3a and Hes7 activity (Ishikawa et al. 2004). Unfortunately, *Nkd1* mutant embryos do not show segmentation defects, which makes unlikely its function as an intracellular oscillation synchronizer (Li et al. 2005). Another candidate is the Wnt pathway member *Lef1* gene, which is required for *Dll1* periodic expression. However, the function of Lef1 as synchronizer is compromised by the fact that *Lef1* expression does not oscillate in the PSM (Galceran et al. 2004; Hofmann et al. 2004). Thus, the question about the coupling between the cyclic genes in the Fgf, Notch and Wnt pathways remains unanswered.

## Conversion of a temporal into a spatial pattern

Real-time imaging of *Hes1* expression suggests that a given cell moving from the tail bud undergoes around five oscillations before reaching the S0 somite (see Figure 1(a) for this nomenclature). The mechanism by which the PSM oscillations are converted into a spatial pattern is unknown, but theoretical models have provided with some hints. One possibility is that the PSM gradients affect a biochemical parameter of the segmentation clock by which the oscillation period tends towards infinitive in the most rostral PSM (Kaern et al. 2000). Another possibility is that a permanent record, eg a covalent protein modification, is made when the PSM cells exit the tail bud, and the actual somite formation depends on the time needed to interpret this record (Kerszberg and Wolpert, 2000). Another possibility is that the oscillation arrest is defined after a given number of oscillations, e.g. through the accumulation of some molecule (Jaeger and Goodwin, 2001, 2002).

**Figure 4:**
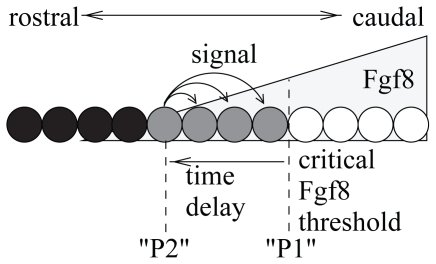
In this model, two PSM positions P_1_ and P_2_ are defined by a Fgf8 threshold value and a time delay, respectively. After reaching the second position P_2_, cells send a diffusive signal to which only cells between the P_1_ and P_2_ (gray cells) are able to respond.

Experimental data suggest that there is a threshold value in the Fgf8 gradient under which PSM cells are able to differentiate into new somites (Dubrulle et al. 2001). To account for this data, some underlying ideas of the cell cycle model have been recently adapted to the clock-and-wavefront model. According to this model, there are two points controlled by the Fgf8 gradient (Figure 4). Cells reaching the second point send a transient signal to which, only cells between both points are able to respond by forming a new somite (Baker et al. 2006).

We speculate that such a transient signal might be encoded by the bHLH gene Mesp2, which periodically appears in the S-1 somite and becomes restricted to the caudal half of the S-1somite (Saga, 2007). Mesp2 is critical for stopping the oscillations and inducing a new segmental border. To achieve this aim, Mesp2 represses Notch signaling in the rostral half of the S-1 somite by inducing *Lfng* expression and inhibiting *Dll1* expression (Morimoto et al. 2005). Unfortunately, the mechanism by which the narrow region of *Mesp2* is defined and whether there is a relation between the Fgf8 threshold value and the Mesp2 activity requires further investigation.

## Conclusions

Current data support the general picture of the clock-and-wavefront, where there is a somitogenesis clock that is progressively arrested to define new somites. However, known mutations do not completely abolish the mouse segmentation, which suggests not only one but several clocks coupled by unknown mechanisms.

In the last years, it has been shown that the dynamics of such clocks depend on specific properties like the transcription/translation delay, short half-life and cooperative binding between repressor and promoter to achieve sustained oscillations. Some of these properties like the half-life have been proved experimentally, but others remain to be proved.

In addition to more experiments, rigorous answers to these questions will require integration and critical testing of current data by the way of mathematical modeling. This combination of experiments and mathematics is a challenge as the collaboration between modelers and experimental biologists is not always easy (Lawrence, 2004). However, as shown here, the strongest evidence arises when experiments and mathematical models are combined, so that we hope this trend to increase in the future.
